# On the generalized fractional integrals of the generalized Mittag-Leffler function

**DOI:** 10.1186/2193-1801-3-198

**Published:** 2014-04-22

**Authors:** Shakeel Ahmed

**Affiliations:** Department of Applied Mathematics, Faculty of Engineering, Aligarh Muslim University, Aligarh, 202002 India

**Keywords:** Generalized Mittag-Leffler Function, Generalized fractional calculus operators, Generalized wright function

## Abstract

**Abstract:**

In this paper, we employ the generalized fractional calculus operators on the generalized Mittag-Leffler function. Some results associated with generalized Wright function are obtained. Recent results of Chaurasia and Pandey are obtained as special cases.

**2000 Mathematics Subject Classification:**

33C45, 47G20, 26A33

## Introduction

In 1903, the Swedish mathematician Mittag-Leffler ( 1903) introduced the function
1.1

where *z* is a complex variable and *ν*≥0. The Mittag-Leffler function is a direct generalization of exponential function to which it reduces for *ν*=1. For 0<*ν*<1 it interpolates between the pure exponential and hypergeometric function  Its importance is realized during the last two decades due to its involvement in the problems of physics, chemistry, biology, engineering and applied sciences. Mittag-Leffler function naturally occurs as the solution of fractional order differential or fractional order integral equation. The generalization of *E*_*ν*_(*z*) was studied by Wiman ( 1905) and he defined the function as
1.2

which is known as Wiman function.

In 1971, Prabhakar ( 1971) introduced the function  in the form of
1.3

where  and  is an entire function of order [ *Re*(*ν*)]^-1^. Special Cases: (i) Setting *δ*=1 in (1.3), we have


(ii) Setting *ρ*=*δ*=1 in (1.3), we have


(iii) Setting *ν*=*ρ*=0 in (1.3), we have


For various properties and other details of (1.3), see (Kilbas et al. 2004).

The generalized Wright function _*p*_*Ψ*_*q*_ defined for  and *α*_*i*_,*β*_*j*_∈*R*(*α*_*i*_,*β*_*j*_≠0;*i*=1,2,...,*p*; *j*=1,2,...,*q*) is given by the the series
1.4

where *Γ*(*z*) is the Euler gamma function ((Erdlyi et al. 1953), Sec. 1.1) and the function (1.4) was introduced by Wright ( 1935) and is known as generalized Wright function. Conditions for the existence of the generalized Wright function (1.4) together with its representation in terms of Mellin-Barnes integral and in terms of H-function were established in (Wright 1934). Some particular cases of generalized Wright function (1.4) were established in (( Wright 1934), Sec. 6). Wright ( 1940a,c) investigated, by “steepest descent” method, the asymptotic expansions of the function *ϕ*(*α*,*β*;*z*) for large values of *z* in the cases *α*>0 and -1<*α*<0, respectively. In Wright ( 1940c) indicated the application of the obtained results to the asymptotic theory of partitions. In (Wright [Bibr CR13][Bibr CR14],b) Wright extended the last result to the generalized Wright function (1.4) and proved several theorems on the asymptotic expansion of generalized Wright function _*p*_*Ψ*_*q*_(*z*) for all values of the argument *z* under the condition,
1.5

For a detailed study of various properties, generalizations and applications of Wright function and generalized Wright function, we refer to papers of Wright ( [Bibr CR12][Bibr CR13][Bibr CR14],b,c) and Kilbas ( 2002)

## Fractional calculus operators and generalized fractional calculus operators

The left and right-sided Rimann-Liouville fractional calculus operators are defined by Samko et al. ( 1993), Sec. 5.1. For *α*∈*C*(*Re*(*α*)>0)
2.12.22.32.4

where [*α*] means the maximal integer not exceeding *α* and {*α*} is the fractional part of *α*.

An interesting and useful generalizations of the Riemann-Liouville and Erdlyi-Kober fractional integral operators has been introduced by Saigo ( 1978) in terms of Gauss hypergeometric function as given below. Let *α*,*β*,*γ*∈*C* and *x*∈*R*_+_, then the generalized fractional integration and fractional differentiation operators associated with Gauss hypergeometric function are defined as follows:
2.52.62.72.8

Operators (2.5)-(2.8) reduce to that in (2.1)-(2.4) as follows:
2.92.102.112.12

Here, we also need the basic result given below (see Rainville ( 1960), Theorem 18, p. 49.)

### **Lemma1**

If Re(c-a-b)>0 and if *c* is neither zero nor a negative integer, then
2.13

## Left-sided generalized fractional integration of generalized Mittag-Leffler function

In this section we consider the left-sided generalized fractional integration formula of the generalized Mittag-Leffler function.

### **Theorem1**.

If , *Re*(*α*)>0, *Re*(*ρ*+*γ*-*β*)>0,*ν*>0,*λ*>0, and *a*∈*R*. If the condition (1.5) is satisfied and  be the left-sided operator of generalized fractional integration associated with Gauss hypergeometric function, then there holds the following formula:
3.1

### Proof

Denote L.H.S. of Theorem 1 by *Ω*, then


Using the definition of generalized Mittag-Leffler function (1.3) and fractional integral formula (2.5), we get


By using Gauss hypergeometric series ((Srivastava and Karlsson 1985), p.18, Eq. 17), series form of generalized Mittag-Leffler function (1.3), interchanging the order of integration and summations and evaluating the inner integral by the use of the known formula of Beta Integral and finally by the use of above lemma, we have


or


which completes the proof. □

### **Corollary1**

If , *Re*(*α*)>0, *Re*(*ρ*+*γ*-*β*)>0,*ν*>0,*λ*>0, *a*∈*R* and if the condition (1.5) is satisfied and  be the left-sided operator of generalized fractional integration associated with Gauss hypergeometric function, then there holds the following formula:
3.2

### **Remark1**

If we set *λ*=*ν* in our result (3.1), we arrive at the result ((Chaurasia and Pandey 2010), (3.1)) given by Chaurasia and Pandey.

## Right-sided generalized fractional integration of generalized Mittag-Leffler function

In this section we consider the left-sided generalized fractional integration formula of the generalized Mittag-Leffler function.

### **Theorem2**.

If , *Re*(*α*)>0, *Re*(*α*+*ρ*)>*max*[ -*Re*(*β*),-*Re*(*γ*)] with the conditions *Re*(*β*)≠*Re*(*γ*),*ν*>0,*λ*>0 and *a*∈*R*. If the condition (1.5) is satisfied and  be the right-sided operator of generalized fractional integration associated with Gauss hypergeometric function, then there holds the following formula:
4.1

### *Proof*.

Denote L.H.S. of Theorem 2 by *I*_2_, then


Using the definition of generalized Mittag-Leffler function (1.3) generalized fractional integral formula (2.6) and proceeding similarly to the proof of theorem 1, we have


or


□

### **Corollary2**.

If , *Re*(*α*)>0, *Re*(*α*+*ρ*)>*max*[-*Re*(*β*),-*Re*(*γ*)] with the conditions *Re*(*β*)≠*Re*(*γ*),*ν*>0,*λ*>0 and *a*∈*R*. If the condition (1.5) is satisfied and  be the right-sided operator of generalized fractional integration associated with Gauss hypergeometric function, then there holds the following formula:
4.2

### **Remark2**.

If we set *λ*=*ν* in our result (4.1), we arrive at the result ((Chaurasia and Pandey 2010), (4.1)) given by Chaurasia and Pandey.

## Left-sided generalized fractional differentiation of generalized Mittag-Leffler function

In this section we consider the left-sided generalized fractional differentiation formula of the generalized Mittag-Leffler function.

### **Theorem3**.

If , *Re*(*α*)>0, *Re*(*ρ*+*β*+*γ*)>0,*ν*>0,*λ*>0, and *a*∈*R*. If the condition (1.5) is satisfied and  be the left-sided operator of generalized fractional differentiation associated with Gauss hypergeometric function, then there holds the following formula:
5.1

### *Proof*.

Denote L.H.S. of Theorem 3 by *I*_3_, then


Using the definition of generalized Mittag-Leffler function (1.3) and fractional differentiation formula (2.7), we have


□

### **Corollary3**.

If , *Re*(*α*)>0, *Re*(*ρ*+*β*+*γ*)>0,*ν*>0,*λ*>0, and *a*∈*R*. If the condition (1.5) is satisfied, then there holds the following formula:
5.2

### **Remark3**.

If we set *λ*=*ν* in our result (5.1), we arrive at the result ((Chaurasia and Pandey 2010), (5.1)) given by Chaurasia and Pandey.

## Right-sided generalized fractional differentiation of generalized Mittag-Leffler function

In this section we consider the left-sided generalized fractional differentation formula of the generalized Mittag-Leffler function.

### **Theorem4**.

If , *Re*(*α*)>0, *Re*(*ρ*)>*max*[*Re*(*α*+*β*+*k*,-*Re*(*γ*)],*ν*>0,*λ*>0, and *a*∈*R* with *Re*(*α*+*β*+*γ*)+*k*≠0 (where *k*= [ *Re*(*α*)]+1). If the condition (1.5) is satisfied and  be the right-sided operator of generalized fractional differentiation associated with Gauss hypergeometric function, then there holds the following formula:
6.1

### *Proof*.

Denote L.H.S. of Theorem 4 by *I*_4_,then


Using the definition of generalized Mittag-Leffler function (1.3) and fractional differentation formula (2.8), we have


□

### **Remark4**

If we set *λ*=*ν* in our result (6.1), we arrive at the result ((Chaurasia and Pandey 2010), (6.1)) given by Chaurasia and Pandey.
